# Extrahepatic cholangiocarcinoma with prolonged survival: a case report

**DOI:** 10.1186/s13256-017-1519-5

**Published:** 2017-12-25

**Authors:** Mohammed Z. Al-Zahir, Turki AlAmeel

**Affiliations:** Department of Medicine, King Fahad Specialist Hospital-Dammam, Dammam, Saudi Arabia

**Keywords:** Cholangiocarcinoma, Klatskin’s tumor, Survival, Mortality

## Abstract

**Background:**

Cholangiocarcinoma has poor prognosis and short term-survival. Here, we report the case of a patient with unusually prolonged survival.

**Case presentation:**

Our patient was a 56-year-old Arab man with a 6-month history of obstructive jaundice. A computed tomography scan of his abdomen revealed a mass at the confluence of the hepatic ducts with suspected malignant strictures on endoscopy. A positive tissue diagnosis was achieved more than 18 months after commencement of his symptoms. He remained functional throughout this period despite recurrent episodes of cholangitis.

**Conclusions:**

Cholangiocarcinoma is a presumably fatal disease, especially because patients tend to present late with unresectable disease. Many patient-related and disease-related factors may alter survival.

## Background

Cholangiocarcinoma is a rare cancer that arises from the epithelial cells of the biliary ducts. It has poor prognosis and short-term survival. Typically, patients present with obstructive jaundice and associated complications of cholangitis and biliary sepsis. Risk factors include conditions with chronic inflammation such as primary sclerosing cholangitis, inflammatory bowel disease, cirrhosis, hepatitis B, hepatitis C, diabetes, and smoking [1–5]. Median overall survival is 20–28 months and 5-year survival rates are as low as 25% [6, 7].

## Case presentation

**Fig. 1:**
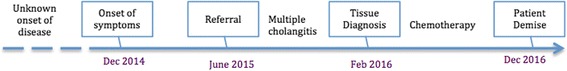
Timeline of events

We report the case of a 56-year-old Arab man who presented to a local hospital with a 6-month history of jaundice, dark urine, pale stool, severe pruritus, and significant weight loss. His medical history was unremarkable. He had a 50 pack-year smoking history (Fig. 1).

His liver biochemistry at presentation was: total bilirubin, 391 umol/L; direct bilirubin, 329 umol/L; alkaline phosphatase, 89 U/L; alanine aminotransferase, 89 U/L; aspartate aminotransferase, 99 U/L; gamma-glutamyl transpeptidase, 222 U/L. cancer antigen 19-9 (CA 19-9), 14 U/mL. his hepatitis B and C screening and autoimmune profile were negative.

**Fig. 2:**
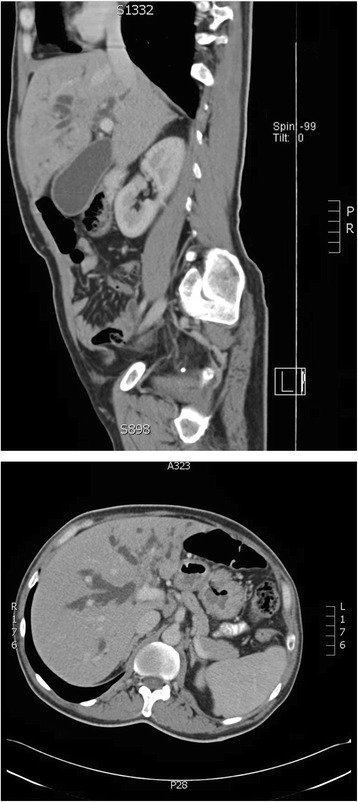
Computed tomography scan of the abdomen, sagittal (*top image*) and axial (*bottom image*) views showing a mass lesion which involved the confluence of the bile ducts extending into both hepatic ducts predominantly affecting the left duct and associated with proximal ductal dilatation

A liver ultrasound scan showed mild hepatomegaly with a dilated common bile duct and intrahepatic biliary radicals. A computed tomography (CT) scan of the abdomen showed a mass lesion involving the confluence of the bile ducts with obliteration of the left portal vein and few porta hepatis lymph nodes (Fig. 2).

A trial of endoscopic retrograde cholangiopancreatography (ERCP) with insertion of a plastic stent was done in the referring hospital. However, good biliary drainage could not be achieved because of biliary strictures.

**Fig. 3:**
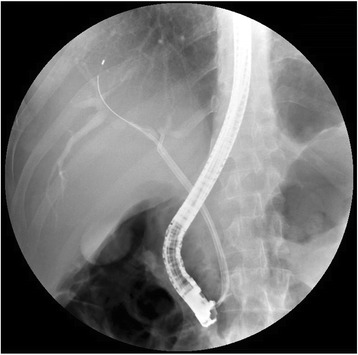
Endoscopic retrograde cholangiopancreatography with wires advanced in the right and the left hepatic ducts

He was referred to our hospital for management of a possibly malignant biliary stricture. At the time of referral, another ERCP trial was done with insertion of two plastic stents and achievement of good biliary drainage (Fig. 3). Brush cytology obtained from the common hepatic duct stricture at the time of the procedure was negative for malignancy.

A month later, a stent exchange was done. Endoscopic ultrasound (EUS)-guided fine-needle aspiration (FNA) of the porta heptais lymph node through the duodenum yielded hyperplastic inflammatory cells. Repeated brush cytology was also negative.

**Fig. 4:**
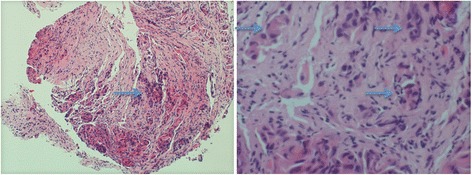
Light microscopic slide stained by hematoxylin and eosin stain, ×10 magnification (*left panel*), ×40 magnification (*right panel*). *Arrows* indicate malignant cells

Then, he was readmitted twice with cholangitis at 3 months and 6 months after the stent exchange. On the latter admission, biliary duct brush cytology was obtained during ERCP that showed histopathological features suggestive of moderately differentiated adenocarcinoma. Immunohistochemical stains showed the following profile: CK-7, positive; CK-9, negative; CA 19-9, positive (Fig. 4). This was 8 months after the initial referral, that is, approximately 18 months after his original presentation.

Our patient was evaluated by hepatobiliary surgeons who concluded that his cancer was inoperable and thus he was referred to medical oncology for further management. A computed tomography scan of his chest, abdomen, and pelvis was done and showed no evidence of distant metastasis. He was started on six cycles of cisplatin-gemcitabine chemotherapy. An end-of-treatment CT scan showed further progression of his disease with newly developed multiple hepatic focal lesions suggestive of metastases versus abscess formation. It was then decided to move him onto palliative capecitabine and he received six cycles till he passed away with an episode of cholangitis. Throughout the course of his illness and despite having recurrent cholangitis and receiving 12 cycles of chemotherapy, our patient remained fully functional till he died from his disease, that is, more than 26 months from his initial presentation.

## Discussion

Cholangiocarcinoma is a presumably fatal disease, especially because most patients present late with unresectable disease. The difficulty in obtaining a tissue diagnosis and presence of mimickers in imaging may delay the diagnosis and, therefore, the commencement of treatment. The main mimicker in multiphasic CT is hepatocellular carcinoma that is differentiated from peripheral intrahepatic cholangiocarcinomas by different enhancement patterns. However, if classic enhancing features are not present, it will be difficult to differentiate them [8].

Different series showing that the overall survival in patients with cholangiocarcinoma is low especially in patients with cholangiocarcinoma of perihilar origin. The 5-year survival for patients with extrahepatic, intrahepatic and distal origin was 10%, 40%, and 23% and the median survival was 13, 28, and 18 months, respectively [9]. In patients who were treated with endoscopic stenting alone the median survival was as low as 8.5 months [10]. In our patient’s case, who presented with advanced inoperable cholangiocarcinoma, he survived for more than 18 months prior to tissue diagnosis and initiation of chemotherapy with full functional capacity despite multiple admissions for recurrent cholangitis. Apart from being male and a smoker, he had no other risk factors for unfavorable outcomes.

Other factors that are associated with improved survival in our patient include high health-related quality of life at presentation, the achievement of satisfactory combined biliary drainage, lack of local or distant metastases, and the hilar origin of the tumor, which is less fatal than the intrahepatic variants [11–13].

## Conclusions

Many patient-related and disease-related factors may alter survival in this fatal disease.
